# Toxicity of Nd_2_WO_6_ nanoparticles to the microalga *Dunaliella salina*: synthesis of nanoparticles and investigation of their impact on microalgae

**DOI:** 10.1039/d1ra04878c

**Published:** 2021-08-10

**Authors:** Mohammad Hassanpour, Seyed Ali Hosseini Tafreshi, Omid Amiri, Masood Hamadanian, Masoud Salavati-Niasari

**Affiliations:** Institute of Nano Science and Nano Technology, University of Kashan P. O. Box 87317-51167 Kashan I. R. Iran Salavati@kashanu.ac.ir hamadani@kashanu.ac.ir +98 31 55913201 +98 31 5591 2383; Department of Biotechnology, Faculty of Chemistry, University of Kashan Kashan I. R. Iran sahosseini@kashanu.ac.ir +98 31 55913201 +98 31 5591 2383; Department of Chemistry, College of Science, University of Raparin Rania Kurdistan Region Iraq

## Abstract

The presence of nanoparticles in the environment and their impact on existing organisms is one of the main concerns of researchers working in this field. In this research, Nd_2_WO_6_ nanoparticles were prepared by an ultrasonic procedure for the first time. X-ray powder diffraction (XRD), Fourier-transform infrared spectroscopy (FT-IR), and energy-dispersive X-ray spectroscopy (EDS) analyses were applied to identify and prove the purity of these particles. In addition to increasing the reaction rate and efficiency with the help of a radical generation mechanism, ultrasound was able to aid the synthesis of these particles. After confirming nanoparticle formation, the optimal nanoparticles in view of scale and morphology were selected by scanning electron microscopy (SEM) and transmission electron microscopy (TEM). Optimal particles at three concentrations (25, 50, and 100 ppm) were mixed into the algae growth medium to investigate the effects of the nanoparticles on *Dunaliella salina* growth. Biological parameters, including the number of cells, biomass, specific growth rate, pigments, and malondialdehyde (MDA), were measured after ten days. Growth parameters showed an increasing trend in concentrations up to 50 ppm; however, at a concentration of 100 ppm, a significant decrease was observed in contrast to the nanoparticles-free treatment. The MDA content showed a linear relationship with enhanced concentration of the nanoparticles. The examination of biological parameters showed that the algae response to stress was dependent on the concentration of nanoparticles. The results showed that 50 ppm of nanoparticles are suitable for increasing algae and achieving a suitable growth rate for commercial purposes. However, in higher concentrations, algal growth inhibition occurs, which is of great importance from a biotechnological point of view.

## Abstract 2

In this work, Nd_2_WO_6_ nanoparticles were synthesized by the ultrasonic method for the first time. Also, for the first time, the impact of these nanoparticles on the environment was investigated.

## Graphical abstract

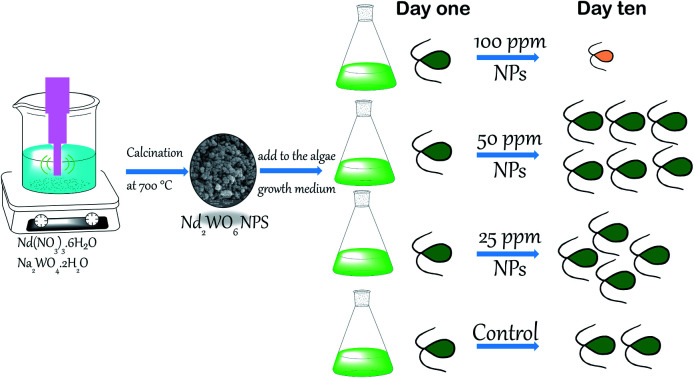

## 1. Introduction

The tungstate family is one of the most broadly used families of nanoparticles. Tungsten-containing metal oxides have potential applications in many fields.^1^ Many applications have been reported for these compounds, such as catalysts, photocatalysts, electrochemical sensors, lasers, supercapacitors, and lithium-ion batteries.^2–6^ Rare metal tungstates and molybdates are well-known hosts for luminescence applications. They are used to fabricate white light-emitting diodes that show high stability, energy savings, and safety.^7^ Chen *et al.* produced Nd_2_WO_6_ particles by mechanical milling due to their dielectric properties and used in the microwave. The reported particle size range was 0.75 to 3.67 μm.^8^ Groń *et al.* prepared Nd_2_WO_6_ particles by the solid-state method with neodymium oxide and tungstate oxide precursors, followed by calcination at 800 °C for 12 hours. In the study of their magnetic behavior, these particles exhibited paramagnetic behavior.^9^

The widespread use of nanomaterials in industry, consumer products, and medicine, their potential for release into the environment, and their effects on ecosystem health have raised concerns about the potential toxicity of nanoparticles to humans and the environment.^10^ Before producing and consuming nanomaterials, nano-researchers must discover and understand their impact on the environment and humans to avoid undesirable properties. Aquatic animals and plants can be used to study the impact of nanoparticles on the environment.^11^ One fascinating type of species to study is the large family of algae.^12^ Among the algal species, *Dunaliella salina* belongs to the family of green microalgae with an egg shape. *Dunaliella salina* is a unicellular biflagellate green microalga belonging to division Chlorophyta, order Chlamydomonadales, and family Dunaliellaceae; its complete scientific name is *Dunaliella salina* (Dunal) Teodoresco 1905.^13^ Due to the adaptation of this species to different conditions and the ability to cultivate it in high quantities, it is of great interest to researchers. On the other hand, *D. salina* can produce carotenoids in large quantities according to environmental conditions. Due to the commercial applications of carotenoids in cosmetics and food supplements, this species is also considered for commercial purposes.^14,15^ This alga can be used to study the effects of nanoparticles on aqueous environments because of its unique properties.

In this work, for the first time, neodymium tungstate nanoparticles were synthesized by the ultrasonic method. Given the application of nanoparticles in various technologies and the possible effects of these materials on the environment, it is essential to study the impact of these materials on aquatic species. In order to achieve two goals, the efficacy of these nanoparticles on *D. salina* alga was perused. The first goal was to achieve a concentration of nanoparticles that inhibited algae growth and was dangerous to the environment. The second was to achieve a concentration of nanoparticles that increased algae growth and subsequently increased the production of valuable pigments for commercial purposes.

## 2. Experimental

### 2.1. Materials

C_2_H_8_N_2_ (en), ammonia, Nd(NO_3_)_3_·6H_2_O and Na_2_WO_4_·2H_2_O, were purchased from Merck. All the mentioned materials were used to prepare nanoparticles.

### 2.2 Preparation Nd_2_WO_6_ nanoparticles

**Scheme 1:**
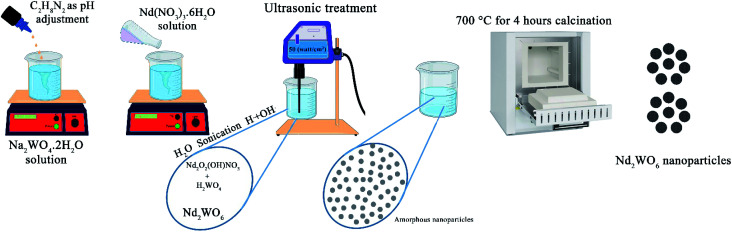
Demonstration of the reaction path.

1 mmol of Na_2_WO_4_·2H_2_O was dissolved in 20 ml distilled water with a magnetic stirrer to synthesize Nd_2_WO_6_ nanoparticles. After that, ethylenediamine (en) as a pH adjustment agent was added to the mentioned transparent solution to set the pH above 7.5. After that, 1 mmol of Nd(NO_3_)_3_·6H_2_O, which was dissolved in 10 ml distilled water separately, was added to the first solution containing tungstate ions. A magnetic stirrer was used to mix the solution. After 5 min, the solution was transferred under sonication (400 W for 5 min). The same procedure was performed for the microwave method (700 W with 1/2 power for 5 min). The gained sediment was washed twice with distilled water and dried at 60 °C for 2 hours. The precipitate was calcined at 700 °C for 4 hours to obtain a suitable crystalline sample. In Table 1, all situations of the experiments are mentioned, and the reaction path is shown in Scheme 1.

**Table 1:** Various synthesis conditions used to synthesize the nanoparticles^a^

No.	Method	Time (min)	Power of irradiation (watt cm^−2^)	pH adjustment agent	Calcination temperature (°C)	SEM images
1	Ultrasonic	5	50	NH_3_	700	Fig. 2(a and b)
2	Ultrasonic	5	50	en	700	Fig. 2(c and d)
3	Ultrasonic	10	50	en	700	Fig. 2(e and f)
4	Microwave	5	700 (watt) (cyclic reaction 30 s on/30 s off)	en	700	Fig. 3(a)

a en: Ethylenediamine.

### 2.3 Characterization

A Philips-X'pert pro instrument was used to achieve the X-ray diffraction (XRD) patterns to confirm the formation and type of crystalline phase. A Nicolet Magna-550 spectrometer was employed to achieve Fourier transform infrared (FTIR) spectra with standard KBr pellets to identify functional groups and intermolecular bonds. Assisted scanning electron microscopy (MIRA3 FESEM) was used to study the shape and appearance of the products, and to determine the deep and internal shapes and specifications of the products, a Philips EM208S transmission electron microscope (TEM) was used. GC-2550TG (Teif Gostar Faraz Company, Iran) was used for all chemical analyses. In addition, the light absorbance feature of the nanoparticles was studied with a UV-Vis spectrophotometer (Shimadzu, UV-2550, Japan).

### 2.4. Cell culture and nanoparticles treatment

Johnson's modified medium was used to cultivate and maintain *D. salina* (UTX LB 2538), which was bought from UTEX.^16^ An Erlenmeyer flask with a volume of 1000 ml was used to prepare the stock culture. In this Erlenmeyer flask, 500 ml of the medium was incubated on a rotary shaker at 25 °C. White light was shined on the sample during the whole incubation period, with a photon irradiance of 100 μmol m^−2^ s^−1^. After obtaining the exponential phase (approximately 5 × 10^4^ cells per ml), an equal amount of algae suspension was used to study the effect of the nanoparticles on algae. Three different concentrations of Nd_2_WO_6_ nanoparticles (C1: 25, C2: 50, and C3: 100 ppm) were used for the treatments, and one sample without nanoparticles was used as a control. A similar growth situation was exerted to maintain the culture, and all samples were controlled for ten days. The biochemical and physiological analysis after the end of the growth period was applied for all treatments.

### 2.5. Methods used to calculate biological parameters

The number of algae cells was counted with the assistance of an Olympus light microscope at 400×. To count algal cells under the microscope, a hemocytometer was used. An equal amount of each treatment was taken to calculate the dry biomass; the samples were filtered *via* a pre-weighed 45 mm glass filter, and after rinsing with double distilled water, they were dried in an oven at 70 °C.

The equation *μ* = (ln *N*_2_ − ln *N*_1_)/(*t*_2_ − *t*_1_) was used to calculate the specific growth rate (*μ*) of algae exposed to nanoparticles. In this equation, *N*_1_ is the number of cells counted from the algae at time *t*_1_ (day one), and *N*_2_ is the number of cells measured for each treatment at time *t*_2_ (day ten).^17^

Equations derived from Lichtenthaler were used to investigate the changes in the level of pigments produced by algae, such as chlorophyll and carotenoids.^18^ Finally, the method proposed by Heath and Packer was used to calculate the amount of MDA produced in the growth medium.^19^

### 2.6 Statistical analyses

Two replications were considered for each treatment. One-way analysis of variance (ANOVA) was employed for the statistical analysis. In each series of data obtained from studying the effects of nanoparticles on algae, the mean was compared using Fisher's LSD test (*p* < 0.05) with the assistance of GraphPad Prism software version 8. For hierarchical clustering analysis (HCA),^20^ the ClustVis online system was used. A heatmap function with correlation-based clustering was used to cluster the treatments.

## 3. Results and discussion

In the synthesis of nanomaterials using the ultrasonic method, different mechanisms have been reported in the literature. These mechanisms include the mechanism of reaction progression through the production of radicals created by ultrasonic waves, the self-sufficiency of vesicles in different conditions of wave application, and the formation of hot spots and nanoreactors with high temperature and pressure.^21–23^ In the synthesis of Nd_2_WO_6_ nanoparticles, a radical mechanism may be involved in developing the reaction.^24,25^ The formation path of the nanoparticles is proposed as follows:1
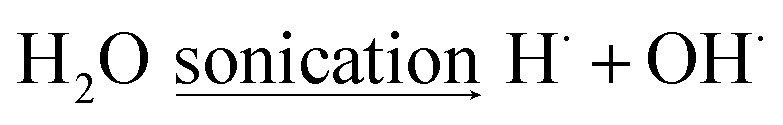

2 Nd(NO_3_)_3_ + OH^−^ → NdONO_3_ + 2NO_3_

3
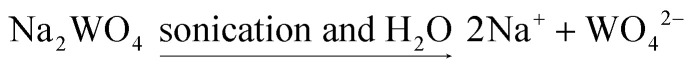

4 2WO42− + 2H+ → H2WO4

5 2NdONO_3_ + H_2_O → Nd_2_O_2_(OH)NO_3_ + H^+^ + NO3−

6 pH < 10.5** **Nd_2_O_2_(OH)NO_3_ + H_2_WO_4_ → Nd_2_WO_6_ + H^+^ + NO3− + H_2_O

**Fig. 1:**
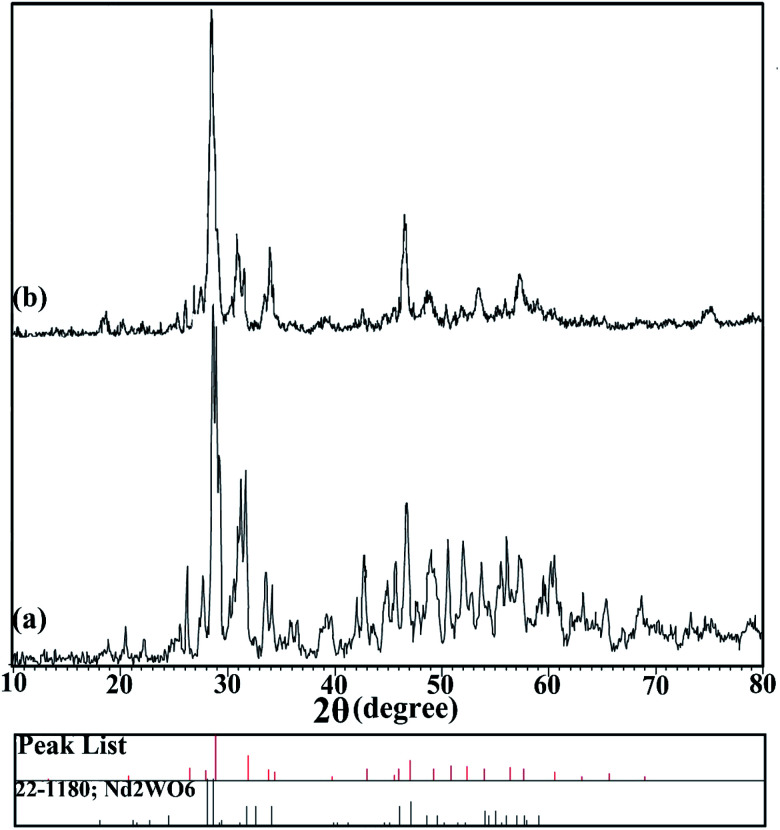
XRD patterns of nanoparticles synthesized (a) by the ultrasonic method (sample no. 3) and (b) by the microwave method (sample no. 4).

X-ray analysis was used to investigate the formation of the nanoparticles. The XRD patterns for the prepared nanoparticles, samples no. 3 and 4, are shown in Fig. 1(a and b), respectively. Fig. 1(a) shows the XRD pattern of the sample prepared in 10 minutes using ultrasonication in the presence of en as a pH-adjusting agent. The index peaks for these nanoparticles at 2*θ* angles of about 31, 33, and 45 degrees are well defined and in accordance with the reference. In comparison, as the pattern shows in Fig. 1(b), for nanoparticles prepared by the microwave method, the peaks became slightly wider. This difference may be due to the smaller size of the particles prepared by the ultrasonic technique, which was investigated by examining the crystallite size using the Scherer equation: *D*_c_ = *Kλ*/*β* cos *θ*.^26^ The crystallite size was about 24 nm for sample no. 3 and about 28 nm for sample no. 4. Both patterns confirm the formation of Nd_2_WO_6_ nanoparticles, and they match with JCPDS 1180-22 and the monoclinic crystalline phase.

**Fig. 2:**
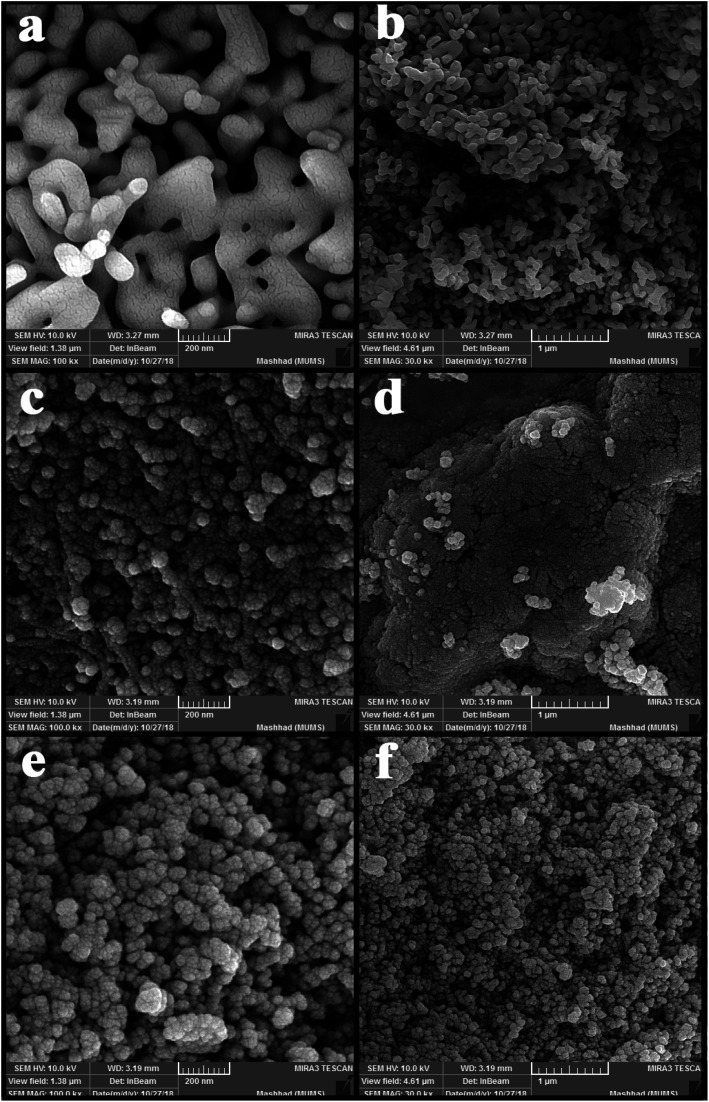
SEM images of nanoparticles synthesized by the ultrasonic method with (a and b) NH_3_ as a pH adjustment agent, (c and d) en as a pH adjustment agent and 5 min under ultrasonication, and (e and f) en as a pH adjustment agent and 10 min under ultrasonication.

**Fig. 3:**
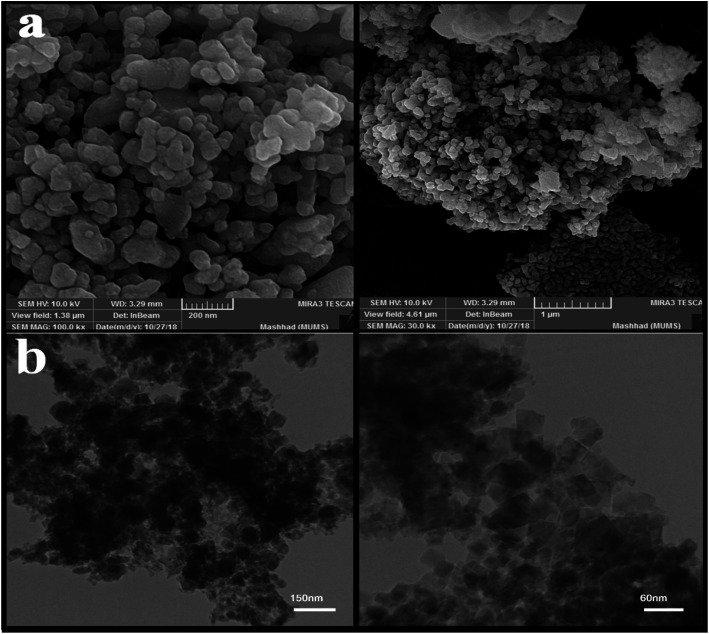
(a) SEM images of nanoparticles obtained from the microwave method, and (b) TEM images of nanoparticle sample no. 3 with magnification.

Fig. 2 shows the SEM images of nanoparticles synthesized at 5 and 10 minutes in the presence of NH_3_ and en as pH-adjusting agents. Fig. 2(a and b) shows nanoparticles synthesized under ultrasonication for 5 min in the presence of NH_3_. As is clear, the particles stuck together to form larger particles. Fig. 2(c and d) and (e and f) show images of nanoparticles synthesized in the presence of en at 5 and 10 min, respectively. At 5 min, the particles are small in size and therefore agglomerate; on the other hand, at 10 minutes, the particles are entirely spherical and well-scattered. Then, the microwave method was applied in pulse mode (700 watts, 30 seconds on, 30 seconds off) to evaluate the contingency of achieving a different shape and size of these nanoparticles. Fig. 3(a) shows the SEM images obtained of the nanoparticles synthesized using the microwave method. Due to the growth of nanoparticles at the time of device shutdown and the predominance of the growth rate relative to nanoparticle nucleation, the particles stick together and form larger particles.

After examining the SEM images and XRD analysis results, sample no. 3 was selected as the optimal sample and used to continue the research.

The image obtained from the TEM analysis is shown in Fig. 3(b) to examine the depth and accuracy of the size and morphology of the nanoparticles in sample no. 3. As shown in the images, the particles are slightly cohesive and aggregated; the size of the nanoparticles is approximately 35 nanometers.

**Fig. 4:**
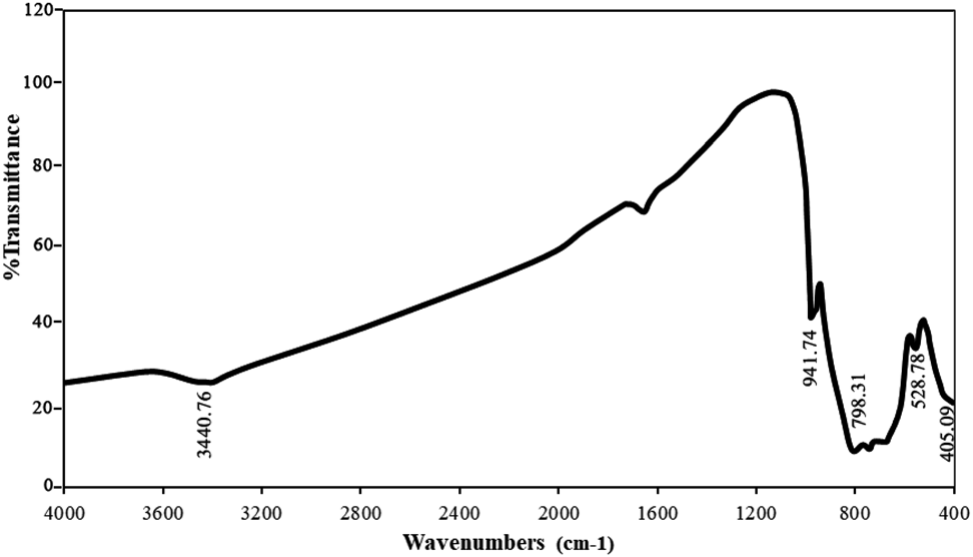
FT-IR analysis obtained from sample no. 3.

The results of the FT-IR analysis (sample no. 3) are shown in Fig. 4. As can be seen, the peaks at 3440 and 1610 cm^−1^ can be attributed to O–H from water absorbed on the surface of nanoparticles. The broad bands at 400–1000 cm^−1^ are attributed to W–O, Nd–O, and W–O–W. The stretching vibrations of Nd–O and W–O are located at 405 and 528 cm^−1^, respectively. Also, the bending vibration modes of W–O–W appeared at 798 and 941 cm^−1^. There is no specific absorption peak in the FTIR spectra, which demonstrates that the as-synthesized Nd_2_WO_6_ nanoparticles are pure.^15,27^

**Fig. 5:**
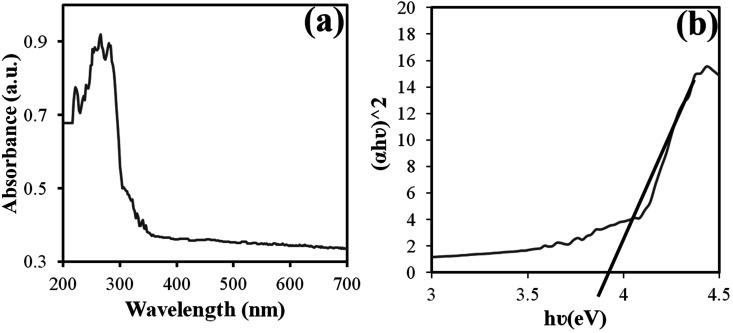
Graphs of (a) DRS and (b) (*αhυ*)^2^*versus hυ* for the band gap estimate obtained from sample no. 3.

DRS analysis at room temperature was used to evaluate the optical properties of the manufactured product (Fig. 5(a)). For materials with direct band energy absorption coefficients, the Tauc equation was used, which is calculated as follows:^28^ (*αhυ*)^*n*^ = *A*(*hυ* − *E*_g_), where *A* is a constant, *E*_g_ is the energy band, and *n* is 1/2 for a direct gap and 2 for an indirect gap. Thus, *E*_g_ is obtained from the linear approximation of the curve (*αhυ*)^2^*versus hυ*. The estimated bandgap for the Nd_2_WO_6_ nanoparticles was approximately 3.9 eV, as shown in Fig. 5(b).

**Fig. 6:**
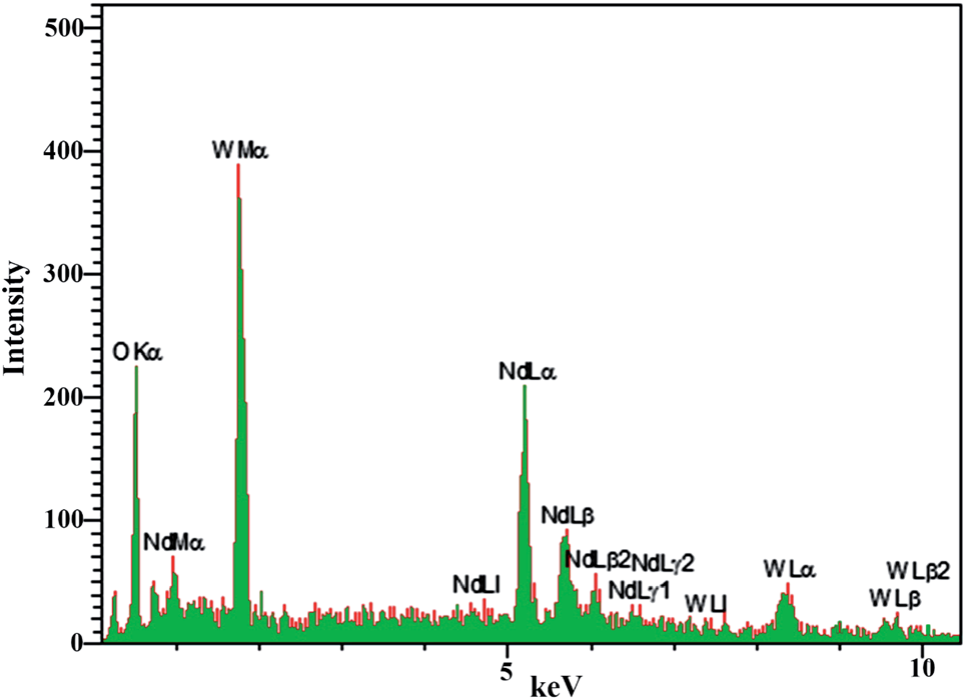
EDS analysis obtained from sample no. 3.

EDS analysis was used to evaluate the purity and absence of various elements in the product. According to Fig. 6, the presence of neodymium, tungsten, and oxygen in the nanoparticles was confirmed by EDS analysis of sample no. 3. Furthermore, the absence of other elements in the spectrum indicates the purity of the nanoparticles.

The optimal nanoparticles of sample no. 3 with three concentrations (25, 50, and 100 ppm) were used to investigate the effects of the nanoparticles on algae. In addition, the free nanoparticles treated with the same conditions were selected as a control sample.

**Fig. 7:**
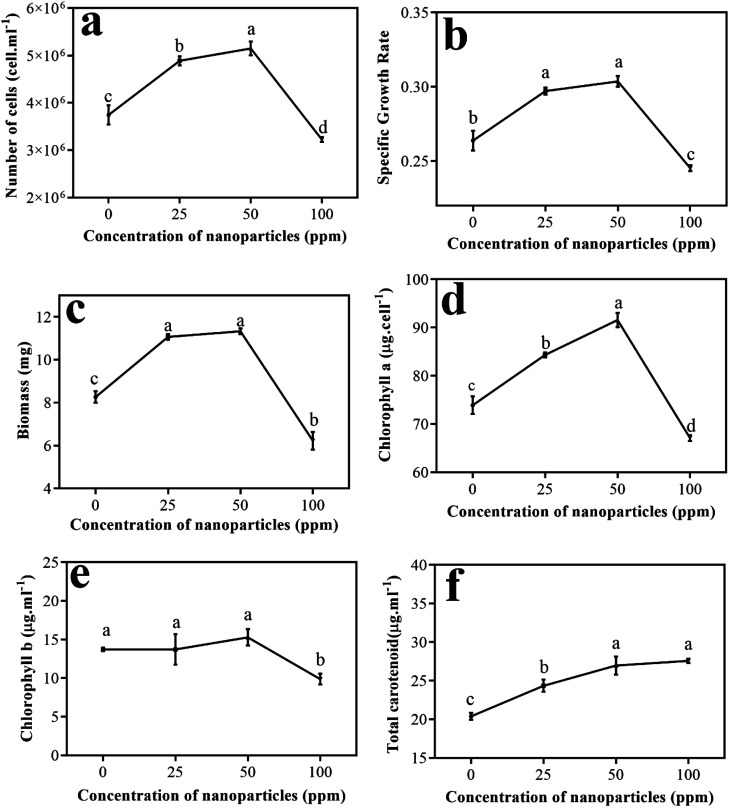
Results of the study of the parameters: (a) number of cells, (b) specific growth rate, (c) biomass, (d) chlorophyll *a*, (e) chlorophyll *b*, and (f) total carotenoids in the presence of different concentrations of nanoparticles.

Fig. 7(a) shows the numbers of algae cells at different concentrations of the nanoparticles. As it is clear, by enhancing the amount of nanoparticles in the environment to a concentration of 50 ppm, the number of cells grew; however, at a concentration of 100 ppm, a significant decrease in the number of algal cells was observed compared to the control sample. This procedure also emerged for the specific growth rate (Fig. 7(b)) and biomass obtained for algae at different concentrations (Fig. 7(c)).

Cao *et al.* studied the effect of doping of CuO nanoscale particles on the recovery of photocatalytic antibacterial activity of graphite carbon nitride for *Microcystis aeruginosa*. In this study, the number of cells obtained during 96 hours at different concentrations (1–100 ppm) showed that there was a significant reduction in the number of cells at a concentration of 100 ppm compared to the control sample. The possible proposed mechanism for this event was that by doping CuO nanoparticles, the heterogeneous accumulation between graphite carbon nitride and algal cells increases, and then reactive oxygen species (ROS) production increases.^29^ Wei *et al.* investigated the effects of different concentrations of SiO_2_ nanoparticles on *Scenedesmus obliquus* algae. They showed that these nanoparticles did not inhibit the growth of the algae at first. They reported that at low concentrations of nanoparticles of about 25 ppm, an increase in growth parameters occurred, but at higher concentrations, the growth trend of the algae changed. Therefore, they considered algal growth to be a function of nanoparticle concentration.^30^

Then, the pigments produced in different treatments were measured. Fig. 7(d) shows a graph of the changes in chlorophyll *a versus* varying concentrations of nanoparticles compared to the control sample. As is evident, chlorophyll *a* has an upward trend up to a concentration of 50 ppm and reaches its peak. Conversely, at a concentration of 100 ppm, compared to the other treatments and the control sample, chlorophyll *a* showed a significant decrease.

Fig. 7(e) shows the graph of chlorophyll *b* levels in different treatments other than the control sample. Although an upward trend is observed from the amount of chlorophyll *b* to a concentration of 50 ppm of nanoparticles, its amount is not remarkably different from the control sample or from the treatment at a concentration of 25 ppm. However, at a concentration of 100 ppm, a considerable reduction in chlorophyll *b* was observed compared to the control sample and other treatments.

Chen *et al.* studied the efficacy of titanium oxide nanoparticles on the algae *Chlamydomonas reinhardtii*. They reported increased ROS due to the presence of TiO_2_, and its attack on chlorophyll caused some chlorophyll *b* to be converted to chlorophyll *a*; therefore, more chlorophyll *a* is seen in cells.^31^ According to this report, the low level of chlorophyll *b* compared to chlorophyll *a* in the sample containing 50 ppm nanoparticles can follow this mechanism. Costa *et al.*, in their study of the effect of Cr_2_O_3_ nanoparticles on *Chlamydomonas reinhardtii* algae, obtained a similar trend for chlorophyll *a* and *b* levels identical to the movement shown by Nd_2_WO_6_ nanoparticles. In this study, by increasing the nanoparticle concentration to 10 ppm, a remarkable reduction in chlorophyll content appeared.^32^

Fig. 7(f) shows the changes in total carotenoid levels for different treatments compared to the control sample. As can be seen, by enhancing the concentration of nanoparticles in the treatments, the carotenoids also increased compared to the control sample. The results of Bahador *et al.* on the impact of silver nanoparticles on *D. salina* showed that beta-carotene content increases under all concentrations of silver nanoparticles compared to the control, which indicates the response of algal cells to the presence of stressors.^33^ Similar phenomena have occurred at different metal stresses in algae, suggesting that the increase in carotenoid synthesis responds to ROS suppression caused by heavy metals.^31^

**Fig. 8:**
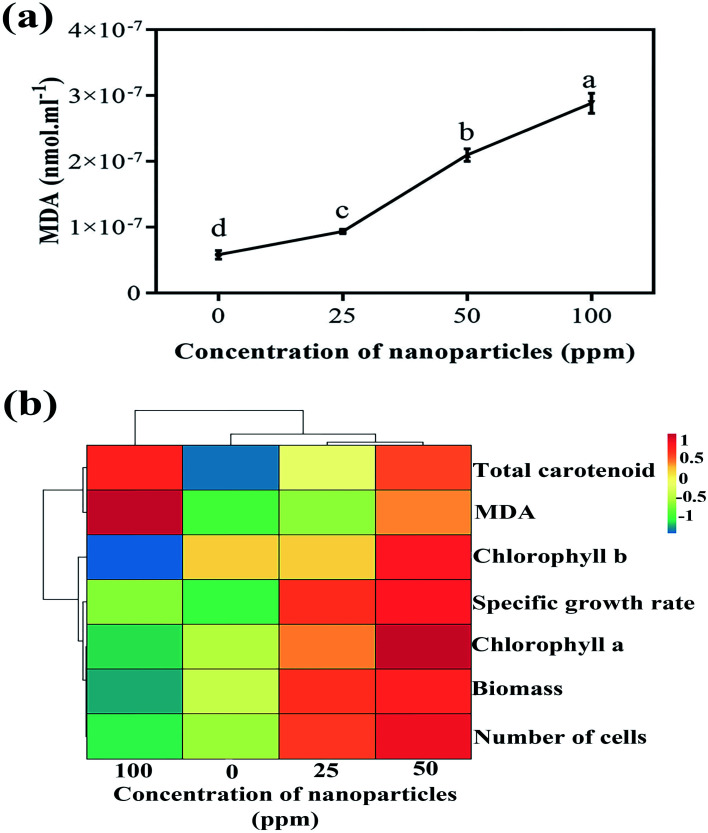
(a) Variations of MDA content at different concentrations and (b) the heat map obtained from the hierarchical cluster analysis of biological parameters.

A direct relationship was observed between increasing nanoparticle concentration and MDA amount (Fig. 8(a)). By enhancing the concentration of nanoparticles in various treatments, the MDA amount also remarkably increased compared to the control sample. MDA is a cytotoxic product and ROS index produced by the oxidation of the algal cell membrane fat layers. There is a positive relationship between the ROS created and the amount of MDA produced. ROS production damages cell membranes through lipid peroxidation, and higher MDA content can be associated with ROS production.^34^ In examining the effect of titanium oxide nanoparticles on the alga *Chlorella pyrenoidosa*, Lin *et al.* showed that the MDA content increased significantly at 20 ppm of concentration of TiO_2_ compared to the control sample and to TiO_2_ concentrations of 5 and 10 ppm.^35^

Fig. 8(b) illustrates the heat map gained from the hierarchical cluster analysis (HCA) of the controlled parameters and different nanoparticle concentrations. As is known, biological parameters are first divided into two general categories; the amounts of MDA and carotenoids are separated from the other parameters. Of course, in the following sub-category, these two parameters are also separated from each other. In the other group, chlorophyll *b* is first isolated from chlorophyll *a*, the specific growth rate, the biomass, and the number of cells. The next subsection separates the specific growth rate from chlorophyll *a*, the biomass, and the number of cells. In the last branch, chlorophyll *a* is separated from the biomass and the number of cells. What can be deduced from this analysis is that if the goal is to increase the growth rate and biomass production of more than algae, a concentration of 50 ppm of nanoparticles is appropriate to achieve this. Due to the reasonable reaction of the algae to an increased concentration of nanoparticles and subsequently increased cell destruction by increasing carotenoid production at a concentration of 100 ppm, this concentration can be used to remove more carotenoids, including beta-carotene.

## 4. Conclusion

According to our results, Nd_2_WO_6_ nanoparticles were synthesized in 10 minutes using ultrasonic waves in the presence of en as a pH adjustment agent. After confirming the formation and selection of the optimal sample in view of scale and morphology by various analyses, three concentrations of nanoparticles were mixed into the algae growth medium. After ten days, the biological parameters of the treatments were measured. The parameter results showed that algae growth at concentrations of 25 and 50 ppm compared to the control sample had an increasing trend, but at a concentration of 100 ppm, inhibition of algal growth occurs. The trends for chlorophyll *a* and *b* were similar. The amounts of carotenoids and MDA showed a linear relationship with the concentration of nanoparticles. With the increase of nanoparticles and, naturally, the rise in ROS, the amount of MDA also increased; on the other hand, algae increased the production of carotenoids to cope with this stress. For commercial purposes, a concentration of 50 ppm nanoparticles can be used to increase algal growth. Also, toxicity and risk to the environment should be assessed and considered when using higher concentrations of these nanoparticles.

## Conflicts of interest

The authors declare that there are no conflicts of interest regarding the publication of this manuscript.

## Supplementary Material
